# The role of self-regulatory control processes in understanding aggressive ideations and behaviors: An experience sampling method study

**DOI:** 10.3389/fpsyt.2022.1058814

**Published:** 2023-01-19

**Authors:** Kerstin Jessica Plessen, Lauriane Constanty, Setareh Ranjbar, Fiorella Turri, Giorgia Miano, Caroline Lepage, Sébastien Urben

**Affiliations:** ^1^Division of Child and Adolescent Psychiatry, Department of Psychiatry, Lausanne University Hospital (CHUV), Lausanne, Switzerland; ^2^Faculty of Biology and Medicine, University of Lausanne, Lausanne, Switzerland; ^3^Center of Psychiatric Epidemiology and Psychopathology, Department of Psychiatry, Lausanne University Hospital, University of Lausanne, Prilly, Switzerland

**Keywords:** ecological momentary assessment (EMA), self-regulation (SR), adolescent, externalizing symptoms, violence

## Abstract

**Introduction:**

In this study, we aimed to examine the association between aggressive ideations and aggressive behaviors in everyday life, as well as the role of processes related to self-regulatory control (i.e., self-control, ego depletion, and emotional states), using experience sampling methods (ESM).

**Methods:**

A total of 62 male adolescents performed a baseline measure of aggression, violent ideations (trait level), and ESM assessments, including four measures per day during nine consecutive days.

**Results:**

At a state level, aggressive ideations were associated with higher negative emotions during the previous day as well as with lower self-control and stronger anger rumination at the moment. Aggressive behaviors were related to higher anger rumination at the moment and to the manifestation of higher intensity of aggressive ideations derived in the previous measure. Higher self-control was related to a lower probability of aggressive behavior.

**Discussion:**

This study highlights the temporal link between aggressive ideations and behaviors in everyday life as well as the role of self-control in understanding aggressivity in the lap of time. Furthermore, we observed that expressions of anger (i.e., reactivity, as well as rumination) were central in the understanding of aggressive ideations and behaviors in the everyday life (i.e., at within-person variability at the state level).

## 1. Introduction

### 1.1. Aggression, adolescence, and psychopathology

During adolescence, individuals face many developmental challenges and, thus, may experience difficulties with emotional regulation which impacts their adjustment and thus their mental health (1). For instance, externalizing symptoms (e.g., antisocial behaviors such as aggression or rule-breaking behaviors) reach a peak during adolescence (2–7). Within this framework, aggression is a central and, depending on the situation, potentially positive element of evolutionary adaptation in humans, emerging in situations in which an environmental stimulus is perceived as a threat, provocation, potential conflict, or due to a personal disposition or both (8). Moreover, violence and aggression also refer to problematic behaviors which entail high costs, both for the individual and the society [e.g., (9–11)]. The health and social burden of violence and aggression are essential. They call for improving our understanding of the emotional and thought processes that accompany the manifestation of violent behaviors or attitudes. Furthermore, it is central to pinpoint the precursors of such behaviors to prevent engagement in those conduct in the long term. In this perspective, aggressive ideations [i.e., thoughts, daydreams, or fantasies of harming someone, either physically, non-physically, or sexually; (12)] represent possible precursors or causal antecedents of aggressive behaviors [e.g., (13–15)].

Little is known about the complex interrelations of aggressive ideations and their relationship to aggressive behaviors over time, especially at the state level or within-person variability. Previous studies observed a positive association between aggressive ideations and corresponding behaviors (16–20). Most of the previous study designs, however, hampered an in-depth understanding of the temporal dynamics and complex interrelationships at a state level by adopting a trait cross-sectional approach. Thus, only the between-person level has been examined, leaving the within-person (or state) level largely unexamined, so far. Several theoretical perspectives reported a long-term causal relationship between aggressive ideations and behaviors (21, 22). In particular, the I^3^ theory (8, 23) seeks to propose a theoretical model giving coherence to the massive number of established risk factors for aggression and future violent behaviors. Indeed, this model evokes three crucial processes: instigation, impellance, and inhibition, which all three influence the likelihood and intensity of a given behavior (i.e., aggressive behaviors) in different ways: the higher the instigation and the impellance and the lower the inhibition, the more likely aggressive behavior may occur. At each stage of the process, the I^3^ model reveals the central role of aggression (21). In particular, instigation describes how the exposition of social dynamics may trigger an urge to behave aggressively. Whereas, impellance reflects the importance of situational factors that psychologically prepare an individual to experience a strong urge to aggress. Finally, inhibition refers to the likelihood that people will override an aggressive urge (e.g., by self-regulation). Instigating and impelling risk factors to combine in a summative manner to establish the strength of the aggressive urge that individuals experience, whereas the capacity of inhibition defines whether an urge results in aggressive behavior or is eliminated in favor of non-aggressive behavior. Applying measures of everyday life, we, thus, examined in an ecologically valid approach the relationships between aggressive ideations and behaviors in adolescents, as well as the role of self-regulation.

### 1.2. Self-regulation

The umbrella term of “*self-regulation*” comprises heterogeneous processes and terms, such as executive functions, effortful control, emotional regulation, or self-control. Self-regulation refers to any intrinsic process that allows an individual to adjust their emotions, thoughts, and behaviors in the ever-changing environment or to achieve long-term goals (24).

Effortful regulation (e.g., emotion regulation or cognitive control) recruits its resources from a pool shared with other effortful processes, which therefore are no longer available for other effortful cognitive or emotional processes [see (25)]. For instance, anger rumination [i.e., unintentional tendency to think about angry experiences; (26)] depletes the limited resources and displays a negative impact on self-regulatory control and may thus facilitate aggressive behavior. Furthermore, depleted resources were proven to drive toward inappropriate behaviors, such as impulsive decision-making [e.g., (27, 28)] or the over-interpretation of ambiguous social cues as threatening (29, 30).

### 1.3. Self-regulatory control and aggression

The deficits of self-regulatory control are hallmarks of externalizing symptoms and in particular of aggressive behaviors (31, 32). When individual experiences emotional states (or arousal) to a degree that seems beyond her/his self-regulatory control capacities (33, 34), which may be due to the strength of the reaction or prior depletion of resources, the individual may behave aggressively (35, 36). Thus, anger may be inappropriately felt and then expressed in form of violence (37).

A longitudinal study (38) explored the interactions between aggressive ideations, aggressive behavior, and self-control (inter-individual variability at the trait level) during 2 years of adolescence. The authors observed that aggressive ideations remained stable at a trait level between 15 and 17 years of age. Likewise, they detected moderate to strong positive interrelationships between aggressive ideations, and aggressive behaviors, as well as a negative association with self-control which emphasizes the availability of limited resources. Moreover, at a state level (experimental induced) it has been shown that aggressive ideations may deplete self-control processes (or cognitive control) and lead to aggressive behaviors. In particular, the cascade effect of aggressive ideations (i.e., angry and revenge thoughts) reduces the individual's resources of self-regulatory control and, consequently, compromises the capacity to control aggressive urges (39, 40).

### 1.4. Intra-individual variability (state level): Experience sampling method

Little is known, however, about the dynamics of the relationships between aggressive ideations and behaviors, as well as the role of processes of self-regulatory control at the intra-individual level (or the state level). Integrating “real-time” within-person approaches, provides important information concerning the symptom variability over time, both at an individual and group level [e.g., (41, 42)]. Indeed, these approaches allow examining changes in cognition and behavior over time, known as dynamic processes at an intra-individual level (43). These dynamic assessments represent an area of development when apprehending the complex interplay between self-regulatory control processes (i.e., emotion states, anger rumination, self-control), aggressive ideations, and behaviors during adolescence and could open new avenues of early intervention.

Experience sampling method (ESM) or ecological momentary assessment (EMA) consists of frequently repeated assessments of thoughts, cognition, experiences, and behaviors in the naturalistic environment in real time, thus allowing to reduce of retrospective recall biases (43) and to assess the temporal sequences among theoretically-linked constructs (44, 45). Moreover, the micro-level approach improves the possibility to observe inferences (46, 47) between the loss of self-regulatory control and the expression of externalizing symptoms (i.e., aggression), allowing in-depth monitoring of the interplay. For instance, Odgers and Russell (48) observed the impact of witnessing violence in young adolescents not only on the same-day measure of psychopathological symptoms (e.g., depressive symptoms, irritability, and conduct problems) but also on the next day. To the best of our knowledge, no previous study has established the temporal interrelationships between aggressive ideations and behaviors at the state or the intra-individual level. Along the same lines, it is not clear how the various processes related to self-regulatory control, namely self-control of impulses, ego depletion, and emotional states, moderate the associations of aggressive ideations with behaviors.

### 1.5. The current study

Previous studies examined only the role of self-regulatory control processes at the between-person level leaving the within-person level largely unexplored. Using the ESM approach will lead to examining the role of self-regulatory control processes at the within-person level in his context of expression (in real life or natural environment) during the important phase of adjustment that adolescence represents. Moreover, such an approach opens the possibility to study the temporal dynamic between the processes in play that have not been covered, so far. We thus aimed to study the role of processes related to self-regulatory control in a naturalistic environment to better understand the link between violent ideations and verbal aggressive behaviors in everyday life through ESM (i.e., taking into account both between-person variability or trait level and within-person variability or state level).

## 2. Method

### 2.1. Sample

**Figure 1:**
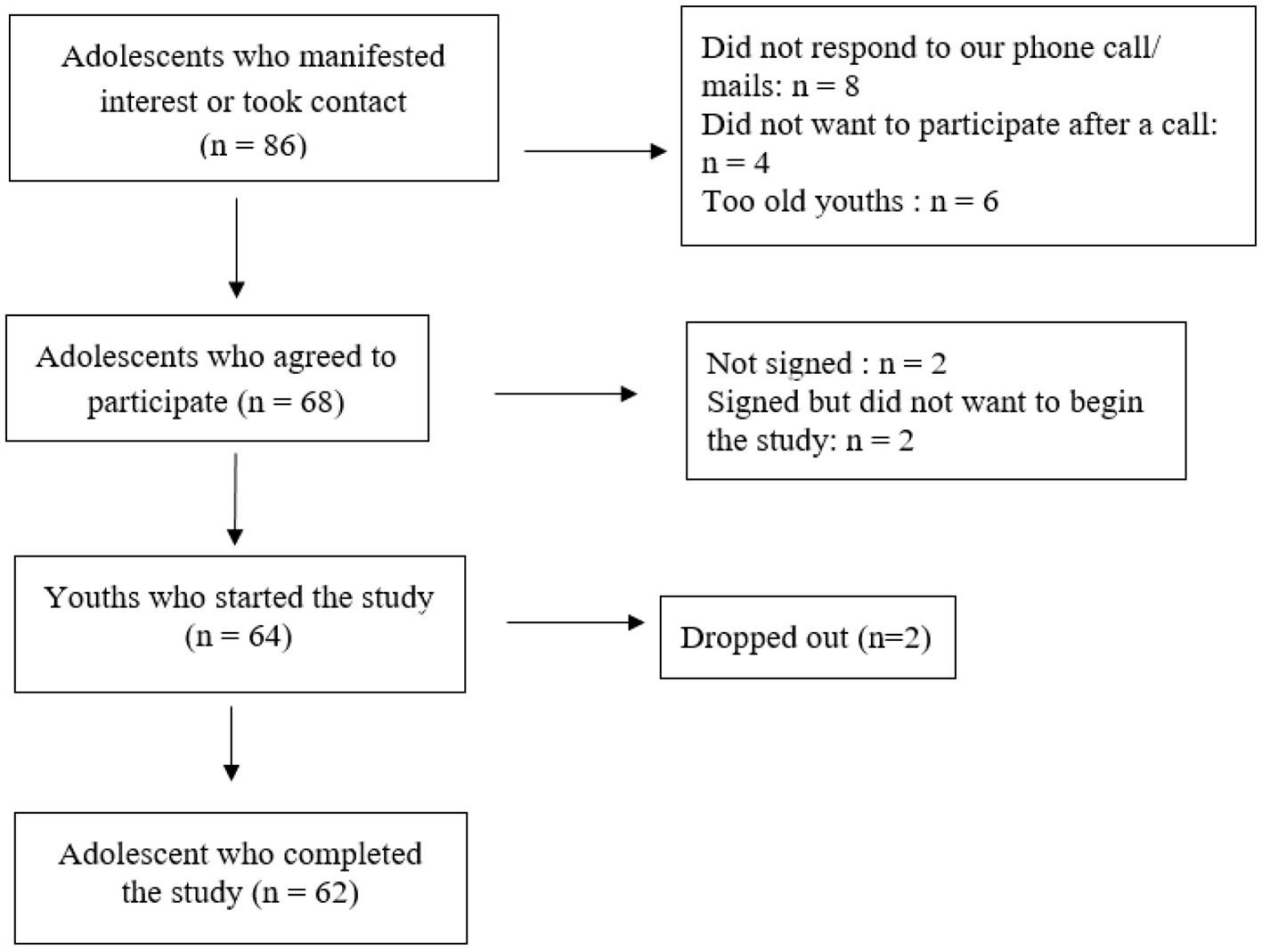
Flow chart.

We included a community sample with a wide range of behavioral and emotional difficulties in a dimensional approach to assure a better apprehension of the interplay of aggressive ideations and behaviors and the role of self-regulatory control processes and implemented a twofold recruitment procedure. First, we advertised for a wide population of the study on dedicated websites (hospital websites and youth-specific websites) as well as at schools. Second, we collaborated with residential care institutions to recruit youths. We included male adolescents aged between 12 and 18 years with a smartphone (Table 1). Potential candidates were excluded if the level of French was insufficient or if they had any known diagnoses of schizophrenia or psychosis or autism spectrum disorders at the present time or were treated with psychotropic medication (i.e., antipsychotic), which has an impact on the behavior and emotion regulation (Figure 1). As this study refers to a pilot study examining the role of self-regulatory control processes at a micro-level, we included only male participants as externalizing problems are more prevalent in boys (49).

**Table 1:** Socio-demographic characteristics (*n* = 62).

**Variable**	**Level**	**M (SD)/% (*n*)**
Age (yrs)		15.19 (1.53)
Socio-economic level	Low	17.24 (10)
	Medium	37.93 (22)
	High	44.83 (26)
Nationality	Swiss	82.25 (51)
First language	French	90.23 (56)
Ongoing education	Yes	95.16 (59)
Education	Primary school	37.10 (23)
	Secondary school	58.06 (36)
	Other^*a*^	4.84 (3)
Living	At home	75.81 (47)
	In institution	20.97 (11)
	Other^*b*^	3.18 (2)

a For instance, tertiary school or apprenticeship.

b For instance, boarding school during the weekday and family during the week and or foster care family.

The majority of participants were Swiss male adolescents (aged 15 years) from high and medium socioeconomic levels, with French as their native language, attending school, and living at home.

### 2.2. Procedure

The study was authorized by the ethics committee of the Vaud state (#2019-02318). After receiving oral and written information about the study, each adolescent and his legal representative agreed in the context of written consent to participate. The study consisted of a baseline assessment (i.e., between-person differences, trait level) followed by an ESM assessment (i.e., within-person variability, state level). The ESM protocol consisted of four measures per day through nine consecutive days (weekends excluded, to assure a homogenous sampling regarding the great variations of daily activities between weekend days and working days). SMS messages prompted the participants to respond to the link to the Redcap^®^ survey. The link was sent at 7 a.m., 12, p.m., 4 p.m., and 8 p.m. The completion of each assessment lasted about 4 min. The participants received a monetary incentive for their participation (100 CHF in total).

Overall, the protocol of the study was properly followed by the participants. All baseline questionnaires were completed for the 62 participants. Furthermore, we collected 2,232 ESM assessments for the 62 participants, with a mean response rate of 85.5% (median = 94.4%, range = 2.8–100.0%). This represents a high compliance with the protocol compared with previous studies using ESM in children and adolescents [for reviews see (50–52)].

### 2.3. Measures

#### 2.3.1. Baseline measures (trait level)

We determined the socioeconomic status (SES) based on the father's and mother's jobs and the highest degree of education based on the Kiddie Schedule for Affective Disorders and Schizophrenia (K-SADS) (53) as SES has been proven to be related to aggression (54, 55). We used the two subtests (i.e., similarities and vocabulary) of the WISC-V (56) to estimate verbal comprehension skills (or verbal intelligent quotient). Then, adolescents rated a series of self-report questionnaires: The Adverse Childhood Experiences (ACEs) scale (57), a 10-item questionnaire (rated as present or not) to encompass various dysfunctional physical and emotional experiences lived in childhood; the Youth Self Report (YSR) from the Child Behavior Check List (CBCL) (58, 59) to rate externalizing symptoms (n = 16 with T-scores ≥ 65) and adjustment problems (n = 9 with T-scores ≥ 65) (60, 61); the Reactive and Proactive Aggression Questionnaire (62, 63) to assess aggression; the Violence Ideations Scale (12), a 12-item instrument designed to provide an assessment of aggressive ideations (i.e., thoughts, day-dreams or fantasies of inflicting harm on another individual); the Brief Self-Control Scale (64) to register trait self-control skills; the General Self-efficacy scale (65), and, finally, the Puberty Development scale (66) to assess puberty.

#### 2.3.2. ESM assessment (state level)

Aggressive ideations were examined through two items (*Since the last assessment…, “…I have thought about smashing someone down because they made me really angry”; “…I have thought about hurting/harming hurt someone I don't like”*) inspired by the violent ideation scale (12). Participants rated their degree of agreement on a slide bar ranging from “*No, not at all*” to “*Yes, totally*” (scoring from 0 to 100; Cronbach's α 0.76). Regarding the skewed distribution, for the analyses we dichotomized the score in the absence (score of 0) or presence (score above 0) of aggressive ideations when this variable was used as the outcome.

We assessed verbal aggressive behaviors through the following item: “*Since the last assessment, I have been verbally aggressive toward someone (e.g., insulted, shouted, said hurtful things)*”. Participants rated their degree of agreement from “*No, not at all*” to “*Yes, totally*” on a slide bar (scoring from 0 to 100). Regarding the skewed distribution, for the analyses, we dichotomized the score in the absence (score of 0) or presence (score above 0) of aggressive behavior when this variable was used as the outcome.

We assessed momentary emotional states by asking, through two items, the participants: “*Now, I feel: nervous/excited*” or “*Now, I feel: angry/frustrated*” they felt rated on a slide bar ranging from “*No, not at all*” to “*Yes, totally*” (scoring from 0 to 100). The degree of nervousness has been rated to ensure the specificity of the role of anger in aggression compared to another negative affect.

We rated the degree of self-control by four questions (*Since the last assessment…, “…I could focus on the ongoing task without being distracted”; …“I could stick with my plans and goals”; …“I have lost control”; …“I resisted to temptation”*). The items were adapted from the effortful subscale of the revised early adolescent temperament questionnaire (67), the Barratt Impulsiveness Scale (68), and the BSCS (64). Participants rated their degree of agreement on the slide bar ranging from “*No, not at all*” to “*Yes, totally*” (scoring from 0 to 100; Cronbach's α 0.66).

We assessed anger rumination with two items (*Since the last assessment…, “…have I ruminated/thought about my past anger experiences?” “…I have analyzed the events that made me angry?”*) adapted from the anger rumination scale (26). Participants rated their degree of agreement on a slide bar ranging from “*No, not at all*” to “*Yes, totally*” (scoring from 0 to 100; Cronbach's α was 0.79.

### 2.4. Statistical analyses

Descriptive statistics characterized the baseline measures of the sample. Mean and standard deviation (SD) were reported for the continuous variables, whereas the number of observations (and their percentages) were reported for categorical variables. First, we performed the Wilcoxon rank sum tests and the Pearson Chi-square tests for continuous and categorical variables, respectively (see Supplementary material for details), to compare the characteristics of those participants who manifested aggressive thoughts (Supplementary Table S1) or behavior and those who did not (Supplementary Table S2) across groups.

Second, we constructed the lag variables to evaluate the association of one measure with the following ones to analyze ideation and behavior in a temporal dimension. Particularly, lag one represented the value of the variable derived from the previous measure (e.g., with the lag one we evaluated the association of a measure in the morning with the midday measure). Whereas, lag four represents the value of the variable derived exactly a day before (e.g., lag four, due to the fact that four measurements took place in 24 h, evaluated the association of the measure in the afternoon of day one with the measure of the afternoon on day 2). We computed the lag variables for all ESM variables.

The two main outcomes of interest aggressive ideations and behavior (i.e., verbally) were the dichotomized version (due to the skewness and the relative infrequency of the presence of aggressive ideations and behaviors, see Supplementary Figure S1) of their continuous measure by ESM at the cutoff zero, indicating if the individual had shown any aggressive ideation or behavior vs. none (i.e., presence or absence).

Finally, we constructed two separate generalized linear mixed effects models with the logit link (69) to investigate the link between aggressive ideations and behaviors. To account for the intraclass correlation of the observation from each participant, a random intercept model was fitted in both models with the individual as the clustering variable. Both preliminary models, first, included all variables and then, we chose to include the variable in the final model, in the function of two criteria, namely the correlation plot (to avoid multicollinearity see Supplementary Figure S2) and the convergence of the model. Therefore, the presented models differed in terms of included variables. Moreover, both multivariate models (on aggressive ideations and behaviors) were adjusted for age and socioeconomic status, as well as self-control, anger rumination, and emotional states derived by ESM and violent ideations from the baseline measurements. The model for aggressive ideations additionally included the state of puberty at baseline, whereas the model for aggressive behavior included the adjustment problem score (assessed through YSR/CBCL). All continuous variables were included in the models as scaled variables using their z-scores. Two measures of goodness of fit were calculated, the “marginal” and “conditional” *R*^2^. The former presents the percentage of the variation explained by the fixed part of the model and the latter the percentage of the variation explained by both fixed and random parts of the model.

We ran the analyses with the data at our disposal without implementing missing data (i.e., case analyses). All analyses were performed using the R environment for statistical computing version 4.1.0 (70). The generalized linear mixed effect model was constructed using the function “glmer” from “lme4” package of R and *p* < 0.05 was considered statistically significant.

## 3. Results

### 3.1. Aggressive ideations

The fitted model with aggressive ideations (state level) as an outcome variable showed that higher anger in the previous day (lag four), lower self-control, and higher anger rumination were significant predictors of a higher probability of aggressive ideations in everyday life [Odd Ratio (OR) =1.21, *p* = 0.014; OR = 0.70, *p* = 0.001, and OR = 1.51, *p* < 0.001, respectively]. Furthermore, a higher level of violent ideations at the baseline (trait level) predicted a higher probability of aggressive ideations at the within-person level (OR = 1.97, *p* = 0.008). The fixed and random effects of the model explained 56.7% of the variation in the outcome as shown by conditional R^2^ (Table 2).

**Table 2:** Generalized linear mixed-effects model on aggressive ideations.

**Level**	**Predictors**	**Odds ratios**	**CI**	***p***
	(Intercept)	1.21	0.35–4.23	0.760
Socio-demo.	Age	1.40	0.70–2.80	0.346
	SES (middle)	0.23	0.05–1.01	0.052
	SES (high)	0.66	0.16–2.74	0.566
Baseline	Violent ideations (VIS)	1.97	1.19–3.26	**0.008**
	State of puberty	0.63	0.32–1.24	0.183
ESM	Anger day before (lag four)	1.21	1.04–1.40	**0.014**
	Self-control	0.70	0.57–0.85	**0.001**
	Anger rumination	1.51	1.28–1.78	<**0.001**
	Nervous	1.05	0.89–1.23	0.568

Marginal R^2^/Conditional R^2^: 0.185/0.567. Socio-demo.: socio-demographic characteristics; ESM: Experience sampling method (state level or every-day life level); SES, Socio-economic status; VIS, Violent ideations scale. Bold: significant factors.

### 3.2. Verbally aggressive behaviors

**Figure 2:**
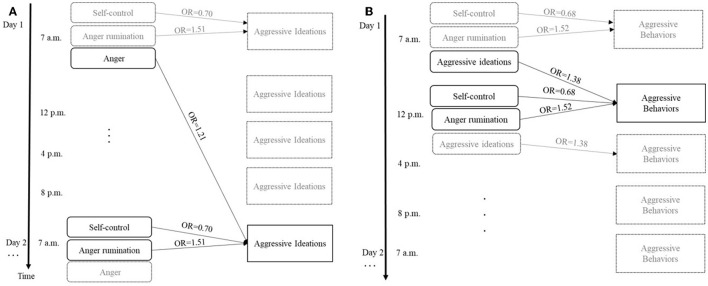
**(A)** Results of the analyses for aggressive ideations **(B)** Results of the analyses for aggressive behaviors. The Figure avoids overlapping arrows, though same pattern applies to every momentary assessment (e.g., aggressive behaviors at 12 p. m. are also associated with the predictors at 12 p. m., etc.). For illustration purpose, only significant results were marked with arrows. OR, Odd Ratio.

More verbally aggressive behaviors during the day of measure (state level) were explained by higher aggressive ideations at the previous measure (OR = 1.38, *p* < 0.001), lower self-control (OR = 0.68, *p* < 0.001), and higher anger rumination (OR = 1.52, *p* < 0.001) (Table 3). In this model, even higher levels of violent ideation at the baseline (trait level) predicted a higher probability of verbally aggressive behavior (OR = 1.58, *p* = 0.046) at the within-person level (Figure 2). A significant difference in the probability of verbally aggressive behavior was predicted by the SES of the youth, showing that participants with medium socio-economic status had a lower tendency to manifest verbally aggressive behavior compared to the lower status (OR = 0.18, *p* = 0.005). In the higher category of SES, only one trend was observed for less verbally aggressive behavior (OR = 0.31, *p* = 0.054) compared with the other two levels of SES. The percentage of the variation explained by both fixed and random parts of the model was 49%.

**Table 3:** Generalized linear mixed-effects model on aggressive behaviors.

**Level**	**Predictors**	**Odd ratios**	**CI**	***p***
	(Intercept)	1.33	0.47–3.75	0.588
Socio-demo.	Age	1.07	0.70–1.62	0.751
	SES (middle)	0.18	0.05–0.60	**0.005**
	SES (high)	0.31	0.09–1.02	0.054
Baseline	Violent ideations (VIS)	1.58	1.01–2.46	**0.046**
	Adjustment problems	0.97	0.59–1.59	0.898
ESM	Aggressive ideations at previous measure (lag one)	1.38	1.16–1.64	**< 0.001**
	Self-control	0.68	0.56–0.82	**< 0.001**
	Anger rumination	1.52	1.30–1.78	**< 0.001**
	Nervous	1.13	0.98–1.31	0.096

Marginal R^2^/Conditional R^2^: 0.199/0.490. Socio-demo.: socio-demographic characteristics; ESM: Experience sampling method (state level or every-day life level); SES, Socio-economic status; VIS, Violent ideations scale. Bold: significant factors.

## 4. Discussion

We explored the interrelationships between aggressive ideations and behaviors in the everyday life of adolescent boys, as well as the moderating role of self-regulatory control processes. To our knowledge, this is the first study adopting an ESM approach with the aim of refining the understanding of the temporal interplay between self-regulatory control processes (i.e., self-control, anger rumination, and emotional states) along with their predictive value in both aggressive ideations and behaviors displayed in everyday life. The presence and the degree of violent ideations and verbally aggressive behaviors were in close relationship to each other at the within-person variability level (or state level), as well as associated with the level of both self-control and anger rumination. In particular, aggressive ideations predicted verbally aggressive behaviors at the next measure (lag one). Moreover, the observed emotional states (i.e., anger) the day before were predictive of aggressive ideations the following day (lag four). On the contrary, verbal intelligence, states of nervousness (another negative emotion), trait adjustment problems, age, trait aggressive behaviors (reactive and proactive), self-efficacy, and adverse life events seem to not influence aggressive ideations and behaviors.

### 4.1. The protective effect of self-regulatory control in the expression of aggressive ideations and behaviors

We observed at a state level that more efficient self-regulatory control processes (i.e., higher self-control and lower anger rumination) predicted a lower likelihood to report violent ideations and verbally aggressive behaviors in general, which is in line with previous literature [e.g., (31, 71–73)]. Moreover, these results are consistent with the fact that self-regulatory control processes permit adaptive responses to both internal and external individual surroundings in the service of long-term goals (24, 74). Taking into account the within-person variability (i.e., state), our results show that high self-regulatory control facilitates the inhibition of verbally aggressive behaviors at moments when impulses of aggression arise.

Equivalently, the results here confirmed and extent that lower self-control, at the state level, predicted an individual's attitude to engage in violent attitudes, both aggressive ideations (39) and aggressive behaviors (38). We observed that these relationships occurred in real time (no observed delays or lag) which may be related to the fact that self-control allows the individual to adapt to the ever-changing environment (24). This role of self-control is accounted for by the I^3^ theory (8, 23), wherein aggression is more likely (and more extreme) when the instigating and pushing forces are strong (producing a strong impulse to attack) and the inhibiting forces (i.e., self-control skills) are weak (producing weak tendencies to overcome the aggressive impulse). In particular, here we observed the impact of the inhibition factor on the avoidance to behave or think aggressively.

### 4.2. Anger rumination, emotional states, and aggressive ideations or behaviors

Furthermore, our results indicated that more anger rumination predicted a higher likelihood of the presence of aggressive ideations and behaviors at a state level. A central feature of anger rumination is overthinking about an event that causes anger (8). This might potentially drive the individual to a state of ego depletion in which the limited self-regulatory control capacities are exhausted and, subsequently, affect the capacity of behavioral responses that rely on self-regulatory control, resulting in maladaptive responses, such as anger and aggression. Aggression as an indicative self-control failure involves the inability to restrain aggressive impulses; although aggressive responding may be more likely when self-regulatory control is diminished, ego depletion alone does not lead to increased aggression (75). In fact, often ego depletion causes an increase in aggression only if preceding provocation or stimulation has occurred, as is the case in rumination (75, 76). Therefore, our findings are in coherence with previous studies, describing that both provocation and self-focused rumination increased the accessibility of aggressive action and arousal cognitions (77).

Moreover, our results revealed, at the state level, the role of emotional states (i.e., anger) as a significant positive predictor only for aggressive ideations the following day. Possibly, emotional arousal spreads functioning like an alarm system, as a result of the salience of negative emotions that dominate the previous memory event and which tend to amplify threats or problems. This emotional reasoning is processed more automatically and quickly than rational judgment, and the reworking of thought processes leads to a search for justifications for feelings rather than testing possible realities (78). This may in turn lead to an increasing degree of aggressive ideas, which confirms the observed preceding negative emotion (e.g., anger) (79). This finding highlights how anger states might represent an important precursor of aggressive ideations, stressing the relevance of the temporal sequences between these processes, as studied here by the application of ESM methodology (44, 45).

### 4.3. Aggressive ideations lead to the manifestation of the related behavior

Furthermore, we disclosed at the state level that anger ideations lead sequentially to verbally aggressive behaviors in the subsequent measure. Similarly, violent ideation as a trait is related to higher aggressive ideations and behaviors in daily life. Thus, angry and vindictive thoughts (i.e., trait and state violent ideations) might limit the fundamental resources for self-control [i.e., ego depletion; (25)] and compromise the individual's self-control functioning (38, 40). Consequently, aggressive thoughts were once drawn out self-control resources may work against the inhibition of aggressive impulses, challenging an individual and temporarily compromising the ability to inhibit aggressive behaviors.

The present findings highlight the importance of the temporal sequences between the “state” processes at play and the possibility of studying them in real-time adds a new context (44, 45). Indeed, we observed that violent ideations represent a precursor that may be measured several hours before the verbally violent behaviors manifest. Moreover, higher states of anger precede violent ideations. This opens new avenues in an intervention such as ecological momentary intervention or just-in-time adapted intervention [see (80)]. For instance, when higher states of anger are detected, we may provide exercises to the adolescent to help manage her/his anger, such as heart-rate variability biofeedback (81, 82) or tools from cognitive-behavioral therapies such as they were demonstrated to be the most effective for anger-related problems among adolescents and adults (83). Moreover, when violent ideations are identified, cognitive restructuring tips may be provided to the adolescents (e.g., how to express their violent ideas appropriately or stressing the negative consequences to behave aggressively). Moreover, to enhance self-control abilities, tools from cognitive remediation programs may be adapted to improve such skills on a daily basis. Within this framework, matching the proposed interventions to the specific deficits observed is essential to increase the benefits (84, 85).

### 4.4. Role of socio-economic status

In line with previous literature (54, 55, 86, 87), we observed that the higher the SES the lower the aggressive behaviors. Lower SES exposed adolescents to a number of risk factors such as parental job loss, poor maternal health, harsh parenting strategies, single-parent households, and poorer quality child care. Such risk factors are related to a greater likelihood to behave aggressively (88). In particular, lower SES exposed the adolescent to more economic stress, which, in turn, is related to more aggression which can be mediated by coping strategies (89). One robust hypothesis to understand the association between low family SES and adolescent aggression posit that children, through social learning, imitate the adult's aggressive behaviors which are normalized (90–92). This hypothesis help us to understand why we observed a link between SES and aggressive behaviors but not with violent ideations (no overt behaviors to mimic).

### 4.5. Limitations

Some limitations of the present study warrant a comment. First, the data rely mainly on self-report measures. Therefore, social desirability bias, as well as a lower insight capacity, may have influenced the results. To reduce this bias, we structured simple and specific questions along with highlighting the importance of honest involvement. Second, we recruited only male adolescents as this pilot study focused on the role of self-regulatory processes in externalizing behaviors through a micro-level approach. This limits the generalization of the results to females and adults. However, this assures a more homogenous sample. Future studies might consider observing a sample of female adolescents, especially taking into account that verbal aggression did not show gender specificity (93) and other age groups to investigate specific patterns in other populations. Third, in our study, we adopted a sampling method represented in a short time period of 9 days. In future studies, a longer sampling period may provide further information and observations on a wider time scale. The advantage of the limited time of data collection was relatively high adherence to the sampling. Finally, we only collected data during the weekdays, which may have had an impact on the results. This was decided to avoid too many confounders in an innovative approach. Further studies, however, should also assess these effects including weekends (when the days are less structured for the youths) to appreciate the differences and complementary effects. We have to dichotomize the data for analytical purposes. Further studies, with larger datasets, may conduct analyses on continuous variables to determine cut off scores where intervention may be needed. We studied how violent ideations lead to verbal aggressivity. Further studies may extend this work to other types of aggression as well to inspect the role of behaviors on ideations.

## 5. Conclusion

In conclusion, the present study highlights the role of self-regulatory control in the understanding of aggressive ideations and behaviors in everyday life. In particular, we outlined the role of self-control among emotional states, and anger rumination in the comprehension of aggressive ideations and behaviors at a state level. These results affine previous knowledge at the trait level, underlining the role of self-regulation in violence [e.g., (31, 73)]. Indeed, our results allowed us to apprehend the temporality of the interplay between the processes as well as to understand the relationships between aggressive ideations and behaviors as well as the precursor effect of anger (i.e., emotional states and rumination). In particular, we observed that the emergence of self-regulatory control processes (i.e., self-control and anger rumination) is important in a given moment, to avoid aggressive thoughts and behaviors. By contrast, anger states (for aggressive ideations) and aggressive ideations (for verbally aggressive behaviors) represent important precursors in the time leading to manifest violence (ideations or behaviors), which open important windows of interventions and reflections for future studies.

Taking into account our results, future studies should include the within-person variability when studying the role of self-regulatory control in violence and develop specific interventions besides already existing treatment [for a scoping review see (94)], especially innovative interventions including naturalistic components [see (80)]. This represents an important line of future research regarding the importance of the different components of self-regulatory control (i.e., self-control, anger states, and rumination) to understand violence and, thus, develop progressively specific interventions to reduce it.

## Data availability statement

The raw data supporting the conclusions of this article will be made available by the authors, without undue reservation.

## Ethics statement

The studies involving human participants were reviewed and approved by the Ethics Committee of the Vaud state (#2019-02318). Written informed consent to participate in this study was provided by the participants' legal guardian/next of kin.

## Author contributions

SU and KP were responsible for the project design, funding, and supervision of the whole study. SU, LC, and GM collected the data. SR conducted the data analyses. SU, SR, and KP interpreted data and drafted different versions of the manuscript. All authors contributed critically to the numerous versions of the manuscript, contributed to the article, approved the submitted version, and agree to be accountable for the content of the work.
