# Supplementary material for: The role of self-regulatory control processes in understanding aggressive ideations and behaviors: An experience sampling method study
Source: Front Psychiatry. 2023 Jan 19;13:1058814. doi: 10.3389/fpsyt.2022.1058814 (PMC9893502; doi:10.3389/fpsyt.2022.1058814)
Supplement: Supplementary file 1 [file Data_Sheet_1.docx]

Supplementary Material

**Figure S1.** Distribution of the outcome variables


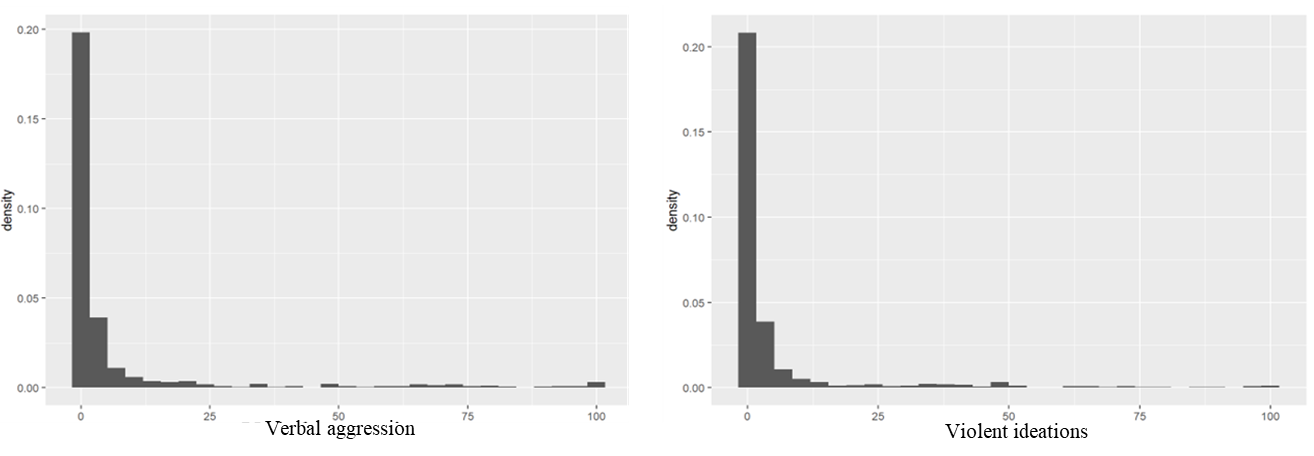


Regarding the skewness of the variables and the relative infrequency of the presence of aggressive ideations and behaviors, we dichotomized in presence and absence for the analyses.

**Descriptive**

The descriptive statistics of the baseline measures in Table S1 and S2 shows that there is no significant age difference between the participants with aggressive ideations or behavior and those with no aggression (mean age 15.14 vs 15.20 years, *p*=0.3). There is a significant difference in the socioeconomic status between the groups with participants with lower socio-economic status have higher probability of showing aggressive behaviour rather than in the second and third categories (*p*<0.001). It is also shown that the group with aggressive ideations and behaviour have significantly (at baseline assessment) higher violent ideation, more trauma, higher aggression (total as well as proactive and reactive) and adjustment problems. The group with aggressive thoughts and behaviour have significantly lower self-control scores as well as self-efficacy scores.

**Table S1.** Comparisons between the goups split by aggressive ideations (from ESM data)

| Characteristic | 0, n = 1,067^1^ | 1, n = 701^1^ | p-value^2^ |
| --- | --- | --- | --- |
| Age (yrs) | 15.13 (1.46) | 15.20 (1.51) | 0.3 |
| SES |  |  | <0.001 |
| Low | 140 (13%) | 133 (19%) |  |
| Middle | 481 (45%) | 231 (33%) |  |
| High | 446 (42%) | 337 (48%) |  |
| Violent ideations | 16.0 (4.3) | 18.5 (6.0) | <0.001 |
| ACE | 0.54 (0.70) | 0.78 (0.98) | <0.001 |
| Aggression (RPQ) | 30.2 (4.3) | 32.4 (4.8) | <0.001 |
| Self control (BSCS) | 41 (7) | 39 (6) | <0.001 |
| Verbal IQ | 11.54 (2.83) | 11.29 (2.31) | 0.10 |
| Self-efficacy | 31.3 (5.3) | 30.4 (4.9) | <0.001 |
| Adjustment problems | 56 (6) | 57 (8) | 0.017 |
| Puberty | 2.78 (0.57) | 2.74 (0.59) | 0.047 |

^1^n from ESM data; Mean (SD); n (%)

^2^Results of Wilcoxon rank sum test or Pearson's Chi-squared test, as appropriate.

Note. SES: Socio-economic status; ACE: Adverse Childhood Event; RPQ: Reactive-Proactive aggression questionnaire, BSCS: the Brief Self-Control Scale; IQ: Intellectual quotient.

**Table S2.** Comparisons between the goups split by aggressive behaviors (from ESM data)

| Characteristic | 0, n = 1,145^1^ | 1, n = 622^1^ | p-value^2^ |
| --- | --- | --- | --- |
| Age (yrs) | 15.14 (1.47) | 15.20 (1.50) | 0.3 |
| SES |  |  | <0.001 |
| Low | 126 (11%) | 146 (23%) |  |
| Middle | 507 (44%) | 205 (33%) |  |
| High | 512 (45%) | 271 (44%) |  |
| Violent ideations | 16.2 (4.4) | 18.5 (6.2) | <0.001 |
| ACE | 0.52 (0.69) | 0.84 (1.01) | <0.001 |
| Aggression (RPQ) | 30.3 (4.1) | 32.6 (5.0) | <0.001 |
| Self control (BSCS) | 41 (7) | 38 (7) | <0.001 |
| Verbal IQ | 11.50 (2.77) | 11.34 (2.39) | 0.3 |
| Self-efficacy | 31.2 (5.3) | 30.4 (4.9) | 0.002 |
| Adjustment problems | 56 (6) | 58 (8) | <0.001 |
| Puberty | 2.77 (0.58) | 2.76 (0.57) | 0.090 |

^1^ n from ESM data; Mean (SD); n (%)

^2^Results of Wilcoxon rank sum test or Pearson's Chi-squared test, as appropriate.

Note. SES: Socio-economic status; ACE: Adverse Childhood Event; RPQ: Reactive-Proactive aggression questionnaire, BSCS: the Brief Self-Control Scale; IQ: Intellectual quotient.

**Correlations**

The correlational analyses help to avoid multicolinearity in the main analytical models.

**Figure S2.** Correlation plot among possible covariates at the between-person level.

**
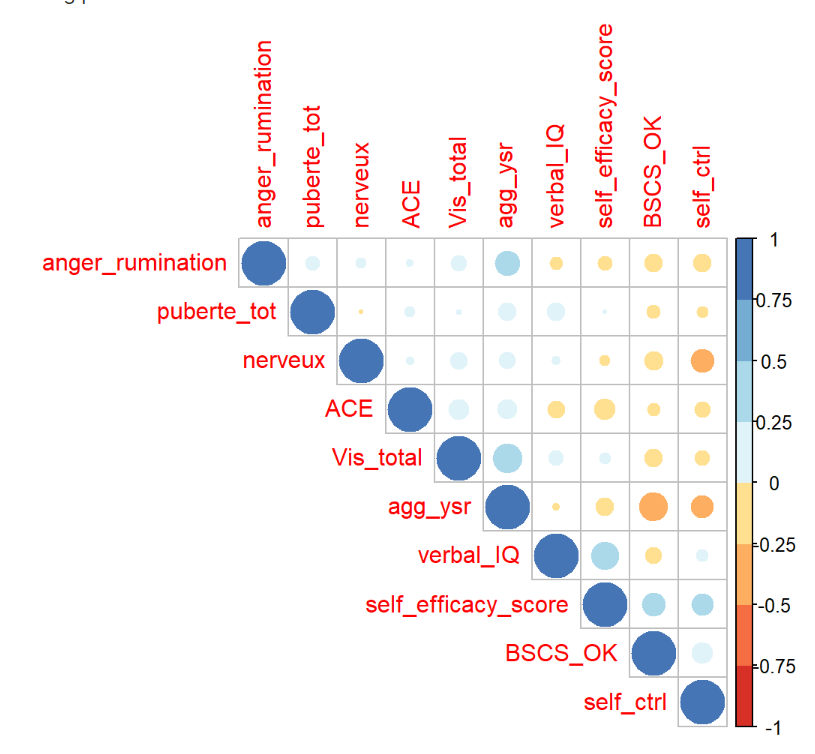
**

*Note*. ACE: Adverse Childhood Event; VIS_total: Violent ideation scale, Agg_ysr: Aggressive scale of the Youths Self-report – CBCL; BSCS: the Brief Self-Control Scale; Verbal_IQ: Verbal Intellectual quotient.

**Table S3.** Description of the full model for aggressive ideations.


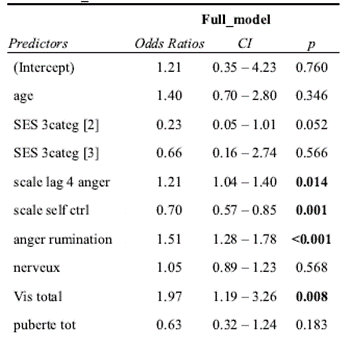


*Note.* SES: socio-economic status, scale lag 4 anger: states of anger from previous day; nerveux: nervousness, VIS total: trait violent ideations, puberte tot : puberty scale.

**Table S4.** Description of the full model for aggressive behaviors.


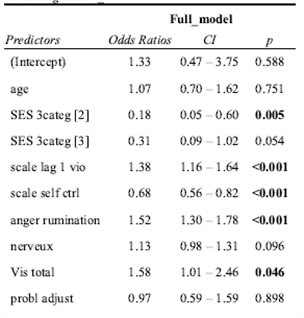


*Note.* SES: socio-economic status, scale lag 1 vio: violent ideations from previous measure; nerveux: nervousness, VIS total: trait violent ideations, probl adjust: adjustment problem from Youth Self-report-CBCL.
